# Treatment of Vaccinia and Cowpox Virus Infections in Mice with CMX001 and ST-246

**DOI:** 10.3390/v2122681

**Published:** 2010-12-13

**Authors:** Debra C. Quenelle, Earl R. Kern

**Affiliations:** Department of Pediatrics, School of Medicine, The University of Alabama, 170 Children’s Harbor Building, 1600 6th Avenue South, Birmingham, Birmingham, AL 35233, USA; E-Mail: ekern@peds.uab.edu

**Keywords:** vaccinia virus, cowpox virus, murine model, orthopoxvirus, antiviral

## Abstract

Although a large number of compounds have been identified with antiviral activity against orthopoxviruses in tissue culture systems, it is highly preferred that these compounds have activity *in vivo* before they can be seriously considered for further development. One of the most commonly used animal models for the confirmation of this activity has been the use of mice infected with either vaccinia or cowpox viruses. These model systems have the advantage that they are relatively inexpensive, readily available and do not require any special containment facilities; therefore, relatively large numbers of compounds can be evaluated *in vivo* for their activity. The two antiviral agents that have progressed from preclinical studies to human safety trials for the treatment of orthopoxvirus infections are the cidofovir analog, CMX001, and an inhibitor of extracellular virus formation, ST-246. These compounds are the ones most likely to be used in the event of a bioterror attack. The purpose of this communication is to review the advantages and disadvantages of using mice infected with vaccinia and cowpox virus as surrogate models for human orthopoxvirus infections and to summarize the activity of CMX001 and ST-246 in these model infections.

## 1 Background and Introduction

Following the tragic events of September 11th and the anthrax mailings in 2001 in the U.S., several laboratories strengthened their ongoing efforts in the discipline of biodefense. Of particular concern was the possibility of an intentional release of smallpox virus as a bioweapon. Funding from government agencies as well as some biotechnology companies was increased and allocated into research and development programs targeted towards the discovery of novel or improved compounds that may be valuable as therapies against orthopoxvirus infections. While more efficacious vaccines for smallpox prevention were also sought and funding provided for that research, the goal of moving at least two new therapeutic compounds into Phase I human clinical trials for safety was of utmost priority. The Project Bioshield Act of 2004 was a multi-billion dollar appropriation made to stockpile both vaccines and therapeutics for use in response to bioterror events. As recently as July 2010, one million doses of smallpox vaccine for certain immune-compromised populations were delivered to the national stockpile from work funded through Project Bioshield (HHS press release, 7/14/10). Two new antiviral drugs, CMX001 and ST-246 are also being considered for inclusion.

Mice have been used extensively for determination of efficacy of antiviral therapies for orthopoxvirus infection and the results have been published as far back as the 1940s [1]. The advantages of using this particular small laboratory animal are numerous. The susceptibility of immunocompetant mice to lethal or non-lethal infections with vaccinia virus or cowpox virus provides an important model system. Lethal models often provide the most definitive and conclusive evidence for antiviral effect. Since laboratory mice are readily available, larger group sizes can be utilized to detect even weakly active compounds and avoid repetitive testing. Chemists can then use that feedback to synthesize more active analogs. The smaller size of the BALB/c weanling mouse in particular, typically less than 15 grams at the initiation of most studies using aerosol or intranasal infections, utilizes small quantities of test compound in concurrent toxicity and efficacy studies. Often, only 50 to 75 mg of an experimental compound is necessary for evaluation. In addition, immunodeficient mice provide models for humans that are immunosuppressed, including post-operative solid organ transplant recipients, leukemia or AIDS patients. To simulate these conditions, severe combined immunodeficient (SCID), athymic, and knock-out mice have been infected with orthopoxviruses and used for antiviral evaluations [2–4].

The disadvantages of rodent models infected with vaccinia or cowpox viruses include the fact that initiation of infection requires a substantial viral inoculum to obtain a lethal infection. This necessity is dissimilar to a smallpox bioterror event where inhalation or contact with only a few airborne infectious viruses to humans could begin a pandemic event [5]. In addition, the pathology of advanced disease in the mouse infected with vaccinia or cowpox virus is not analogous to variola virus related causes of death in human patients which generally result in about a 30% mortality rate, even in naïve, unvaccinated persons. A fatal encephalitis is one cause of death in human patients either post-vaccinal or following acute infection, whereas, the mouse exhibits multi-organ involvement with inflammatory processes and significant lung pathology [6,7]. Mice infected with ectromelia virus require lower infectious doses of virus for initiation of a lethal infection with a pathology that more closely resembles smallpox in humans [8]. A disadvantage of this model is the requirement for more stringent containment procedures which may preclude its use for large scale *in vivo* screening studies.

## 2. Review

Cidofovir (CDV) has been reported to have very good efficacy against orthopoxvirus infections in a number of model systems [3,4,9–11] and has been stockpiled for use in orthopoxvirus infections or complications from vaccination under an investigation new drug protocol [12]. However, practical use of CDV is limited due to the required intravenous route for administration and its dose-limiting nephrotoxicity severely limits its usefulness even in an emergency bioterror or naturally occurring event. CMX001, originally known as hexadecyloxypropyl-cidofovir (HDP-CDV), was only one of several ether-lipid esters of CDV synthesized in a search for compounds that were orally active and had reduced toxicity for use in the treatment of orthopoxvirus and other DNA virus infections [13]. The lipid side chains added to CDV enhanced cellular and oral uptake and altered the biodistribution patterns of CDV which reduced the known nephrotoxicity associated with intravenously administered CDV (Vistide®) [14–16]. The active metabolite, the acyclic nucleoside phosphonate, inhibits viral DNA polymerase independent of viral phosphorylation. A number of nucleoside phosphonates and their analogs were evaluated *in vitro* for their activity against orthopoxviruses and many were significantly more potent than CDV [13,17]. Four of the more active and least toxic ether lipid esters of cidofovir were subsequently tested in mice for toxicity and efficacy against several different vaccinia virus strains: WR, IHD or CDV- resistant -CDV-R [18–20]. Mice infected with cowpox virus, strain BR were also included for similar evaluation [18].

Since CDV was the first and only drug that has been approved for emergency use under an investigational new drug protocol for treatment of an orthopoxvirus infection or adverse vaccine reactions, its efficacy was confirmed in our laboratory using mice infected with either vaccinia or cowpox virus prior to efficacy testing of the new ether lipid esters of CDV. It was also included as a positive control in all experiments used to evaluate the activity of new agents. The *in vitro* activity of CDV and four of the most promising of the ether lipid esters of CDV, hexadecyloxypropyl-CDV (HDP-CDV, CMX001), octadecyloxyethel-CDV (ODE-CDV), oleyloxypropyl-CDV (OLP-CDV), and oleyloxyethyl-CDV (OLE-CDV) against vaccinia virus is shown in Table 1. The four ether lipid esters of cidofovir had effective concentrations (EC_50_ in μM) ranging from 0.8 to 0.06 compared to CDV at 31, a 50-100-fold difference. Clearly all four compounds had greater efficacy than CDV [13]. Their selectivity indices (SI) ranged from 37 to 933 compared to CDV at >10.

The activity of CDV was next evaluated in mice infected intranasally with vaccinia or cowpox virus to determine the essential number of doses, the timing of the doses and the concentrations necessary for improved survival. Since CDV had to be administered i.p. and was already available as an intravenous solution for human use, the highest dose of 100 mg/kg down to the lowest diluted dose of 3 mg/kg were given on multiple days or as a single dose prior to or following lethal infections. As shown in Table 2, even a single dose of CDV administered from five days before viral inoculation to three days post-exposure could significantly (P ≤ 0.05) improve survival of BALB/c mice lethally infected with vaccinia virus. When SCID mice were inoculated i.p. with vaccinia or cowpox virus and treated post-viral infection either daily for seven days or three times weekly for 30 days, there was a significant increase in the mean survival time of animals while on drug. However, upon cessation of treatment all animals eventually died, indicating that drug therapy in the immunocompromised host failed to clear the viral infection. A significant reduction in virus replication was detected in liver, spleen, and kidney, but not lung samples [2].

With the activity of CDV in mice infected with vaccinia and cowpox virus well established in our laboratory, the CDV analogs were then evaluated in these murine models. When the four CDV analogs were given to uninfected mice to determine toxicity, CMX001 given orally on five consecutive days appeared to be the least toxic of the group as measured by mortality [18]. When groups of mice were treated with 5 mg/kg for five consecutive days beginning 24, 48 or 72 h post intranasal inoculation with an LD_90_ dose of vaccinia virus-WR, those treated with CMX001, ODE-CDV or OLE-CDV had improved survival and the results are summarized in Table 3. Similar to the results obtained earlier in SCID mice, animals that were treated with CMX001 or ODE-CDV had titers of virus in their liver, spleen and kidney that were reduced by 3 to 7 log_10_ compared with vehicle-treated mice. Again, no significant reduction of virus replication in lung tissue was observed [18].

Other investigators have reported that when given as a single dose 24 h after infection, CMX001 at 100, 50 or 25 mg/kg improved survival following lethal intranasal infections of mice using a different strain of vaccinia virus, strain IHD [19]. These results are summarized in Table 4. While lower doses of 10 mg/kg or less given over five consecutive days were not effective (Table 4), some toxicity was also documented by decreases in weight gains of uninfected mice that received multiple doses of CMX001 [19]. In subsequent studies using a CDV- resistant strain of vaccinia virus, mice that were intranasally infected with the non-lethal CDV-resistant vaccinia virus-CDV-R and treated with CMX001 at 50 mg/kg p.o. on Days 1 and 3 post-inoculation, had significantly lower lung consolidation scores (0.5 *versus* 2.8) and snout virus titers (4.1 *versus* 5.3) than placebo treated mice [20].

The same four ether lipid esters of cidofovir described above were also evaluated by us using *in vitro* efficacy against cowpox virus strain BR and compared to CDV [18]. Their selectivity indices (SI) ranged from 49 to 800 compared to CDV at >7.5. Their effective concentrations (EC_50_ in μM) ranged from 0.6 to 0.07 compared to CDV at 42 (Table 1). Indeed all four compounds again had greater activity than CDV. Mice treated with a fixed daily oral dose of 6.7 mg/kg for five consecutive days beginning 24, 48 or 72 h post inoculation with cowpox virus had improved survival rates with CMX001, ODE-CDV, OLP-CDV and OLE-CDV as summarized in Table 5.

In summary, orally administered CMX001 was the most effective analog of CDV tested, and proved highly effective in mouse models of orthopoxvirus infections. It was generally as effective as CDV given parenterally.

While CMX001 was an intentional design conceptualized to improve upon the already known antiviral properties of CDV, ST-246 was a uniquely synthesized analogue based on optimization of an active compound detected during large scale, high throughput screening efforts [21]. The effective concentration (EC_50_ in μM) of ST-246 was 0.01 against vaccinia virus -NYCBH and 0.05 against cowpox virus-BR. In these studies, ST-246 had greater efficacy than CDV and inhibited CPE formation more robustly than CDV in cell culture [21]. When evaluated *in vitro* against vaccinia-COP, vaccinia-WR or cowpox-BR viruses in our laboratory, ST-246 also had greater activity than CDV, but had about equivalent potency with CMX001. ST-246 had higher selectivity indices against each virus strain than did CDV or CMX001 due to its reduced toxicity compared with the nucleotides (Table 6). Its mechanism of action is unlike CDV or CMX001 and was reported to affect the extracellular egress of formed viral particles which diminishes viral spread from cell to cell or, as in animal models, into a systemic disease [22].

When ST-246 was given orally to mice at 50 mg/kg twice daily for 14 days following a lethal intranasal infection of vaccinia virus, 100% survival was achieved [21]. Using an alternative model, where mice were injected intravenously using vaccinia virus, ST-246 given orally at 50 or 15 mg/kg twice daily for five days resulted in a dose dependent reduction in tail lesion formation by day 8 post-inoculation [21]. Studies performed in our laboratory evaluated various dosing regimens for efficacy in mice against either vaccinia or cowpox virus [23]. Either longer durations or delays in beginning treatment were required for efficacy of ST-246 against cowpox virus infection in mice, predictably so with the longer term to mortality in the lethal intranasal cowpox virus model of 8–9 days *versus* six days for vaccinia virus as shown in Table 7. Higher doses of 100 mg/kg given orally once daily were generally more effective against mortality from cowpox virus than lower doses when there were delays of treatment initiation of 48 to 72 h (Table 8). When ST-246 was evaluated in immunocompromised animals, it significantly prolonged survival [3], but did not alter mortality indicating that this drug, in the absence of a functional immune system, is also unable to clear virus infection. One important observation regarding ST-246 was a lack of toxicity among various species of animals even when high doses were administered for relatively long periods of time.

Several factors led to our decision to initiate synergy studies with CMX001 and ST-246. First, there was proven efficacy of both CMX001 and ST-246 in small animal models of orthopoxvirus infections. Second, both compounds have been tested in large animal trials using monkeypox or smallpox models. Third, the mechanism of action for each compound was distinctly different and not expected to result in combined toxicities *in vivo*. The benefits of combined therapies would be the ability to use reduced dosages of each compound, reduce the likelihood of the development of resistance and overcome intentionally engineered viruses that had resistance factors for nefarious intent. Additionally, the high level resistance attained with a single point mutation for ST-246 makes the drug highly vulnerable to the development of resistance, but its use in combination requires virus to become resistant to both drugs and effectively raises the genetic barrier of both ST-246 and CMX001.

**Figure 1:**
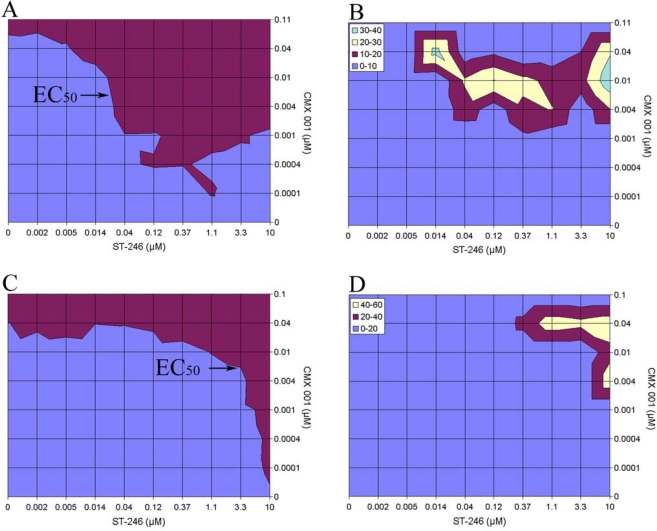
Synergistic interactions of CMX001 and ST-246 against vaccinia and cowpox virus *in vitro*. Effect of combinations of CMX001 and ST-246 against vaccinia virus and cowpox virus. Inhibition of vaccinia virus replication was evaluated in a CellTiter-Glo® assay with a matrix of drug concentrations and an isobologram depicts EC_50_ values at each drug combination (A). A synergy plot is also shown that represents greater than expected inhibition with increasing synergistic intensity represented by maroon, yellow and green regions, respectively (B). This analysis determined that combinations of ST-246 and CMX001 were strongly synergistic with volumes of 326 μM^2^% at the 95% confidence level. Efficacy of this drug combination was also determined against cowpox virus in a neutral red assay and the EC_50_ isobologram is shown (C). A synergy plot also identified several combinations of concentrations where synergistic interactions occurred and are shown at the 65% confidence level (D). This analysis calculated the volume of synergy at 106 μM^2^% at the 95% confidence level. Excerpted from [24].

*In vitro* combination studies using CMX001 and ST-246 were performed against both vaccinia and cowpox virus [24]. While strong synergistic activity was found against vaccinia virus with very low doses across a broad range of combinations, higher concentrations of ST-246 were required for producing similar synergy with cowpox virus (Figure 1). A series of animal studies using combinations of CMX001 with or without ST-246 in cowpox virus-infected mice showed less than anticipated synergy *in vivo* but this may have been due to small numbers of animals and variability in animal to animal pathogenesis of infection. There were modest, but improved, survival rates at suboptimal combination levels when compared to treatment with single agent alone (Table 9) [24]. Five of the groups of mice treated with combinations of CMX001 with ST-246 had reduced mortality (P ≤ 0.01) or increases in mean day to death (P ≤ 0.01) compared to vehicle treated groups when treatments were initiated six days post cowpox virus inoculation.

Both of these antiviral agents have successfully completed human Phase I clinical trials and have been given to a limited number of vaccinia virus-infected patients or in the case of CMX001, other dsDNA virus infected patients under the FDA compassionate use policy with anecdotal successes.

## 3. Conclusions

Mice infected with vaccinia or cowpox viruses have been used to evaluate the efficacy of new antiviral agents for their activity against orthopoxvirus infections in order to minimize the quantities of test compounds required in efficacy testing while providing predictive data for larger species. Inoculations using vaccinia or cowpox virus have utilized the routes of cutaneous, intraperitoneal, intravenous, intranasal and inhalational infection. Mice used in these evaluations have generally been immunocompetant, however, chemically or genetically immunocompromised mice have also been used. There have been several comprehensive reviews in the literature of a variety of antiviral agents tested over the past decade against the orthopoxviruses and their outcomes *in vitro* and *in vivo* [25–27]. That is beyond the scope of this limited review, but numerous publications of peer reviewed research using mice infected with vaccinia or cowpox viruses have been illuminating for the purposes of development of new and more potent antivirals with differing mechanisms of action. Mice have proven to be a prudent and useful tool in antiviral efficacy testing and, most likely, the only financially feasible tool for evaluating combination therapies *in vivo*.

Both antiviral drugs, ST-246 and CMX001, were initially evaluated in mice and proved efficacious and relatively non-toxic at effective levels. Both have progressed into studies using larger animals, including primates infected with monkeypox or variola virus, and have been evaluated for safety in humans through Phase I clinical trials. In addition, both compounds have been given to a limited number of human patients under the FDA’s approval for compassionate use when adverse events followed smallpox vaccinations. Since it is not feasible to conduct large scale Phase III clinical studies for orthopoxvirus infections, neither CMX001 nor ST-246 can be approved for use in treatment of smallpox or monkeypox infections by conventional means. ST-246 has activity only against poxvirus infections and would have to satisfy the requirements of the FDA’s “Animal Rule” to achieve approval. Typically, the FDA requires that efficacy of new compounds be established in both a small animal and large animal model system prior to being considered for human use. In contrast, CMX001 has excellent activity against herpes simplex virus, cytomegalovirus, adenovirus and other DNA viruses; therefore, approval for this drug could be obtained through the conduct of clinical trials against one of these other viruses.

## Figures and Tables

**Table 1:** Antiviral activity and cytotoxicity of ether lipid esters of CDV in human foreskin fibroblast cells.

	**Vaccinia Virus Copenhagen**			**Cowpox Virus Brighton**		

Compound	EC_50_ (μM)^a^	CC_50_ (μM)^a^	SI^b^	EC_50_ (μM)^a^	CC_50_ (μM)^a^	SI^b^
CDV	31 ± 5.4	>317 ± 0	>10	42 ± 5.4	>317 ± 0	>7.5
OLP-CDV	0.4 ± 0.2	87±15	218	0.6 ± 0.3	87±15	145
OLE-CDV	0.06 ± 0.02	56 ± 29	933	0.07 ± 0.02	56 ± 29	800
CMX001	0.8 ± 0.4	31 ± 24	37	0.6 ± 0.3	31 ± 24	53
ODE-CDV	0.2 ± 0.1	14	65	0.3 ± 0.3	14	49

Adapted from [18].

a. Values are the mean of 2 or more assays ± standard deviation.

b. Selectivity Index (SI) = CC_50_/EC_50_;

CC_50_ (concentration causing cytotoxic effect on 50% of uninfected confluent cells); EC_50_ (effective concentration that reduced plaque formation by 50%).

**Table 2:** Effect of single dose CDV on mortality of BALB/c mice inoculated intranasally with vaccinia virus-WR.

**Treatment^a^**		**Mortality**		**P-value**	**MDD^b^**	**P-value**
		**Number**	**Percent**			
Untreated		15/15	100	---	9.1	---
Placebo	Day +1	14/15	93	---	8.6	---
**CDV**						
100 mg/kg	Day −5	1/15	7	<0.001	10.0	0.7
30 mg/kg	Day −5	9/15	60	0.08	9.0	NS
10 mg/kg	Day −5	8/15	53	<0.05	8.8	NS
3 mg/kg	Day −5	14/15	93	NS	8.5	NS
**CDV**						
100 mg/kg	Day −3	2/15	13	<0.001	8.5	NS
30 mg/kg	Day −3	7/15	47	0.01	9.1	NS
10 mg/kg	Day −3	15/15	100	NS	8.4	NS
**CDV**						
30 mg/kg	Day −1	0/15	0	<0.001	---	---
10 mg/kg	Day −1	2/15	13	<0.001	12.0	0.01
3 mg/kg	Day −1	12/15	80	NS	8.6	NS
**CDV**						
30 mg/kg	Day +1	0/15	0	<0.001	---	---
10 mg/kg	Day +1	0/15	0	<0.001	---	---
3 mg/kg	Day +1	4/15	27	<0.001	9.0	NS
**CDV**						
30 mg/kg	Day +3	1/15	7	<0.001	8.0	NS
10 mg/kg	Day +3	0/15	0	<0.001	---	---
3 mg/kg	Day +3	8/15	53	<0.05	8.6	NS

Adapted from [2].

a. Animals were treated one time only for each time period beginning Day −5, −3, or −1 or Day +1 or Day +3 after viral inoculation.

b. MDD = Mean Day of Death.

c. NS = Not significant when compared to the placebo control.

**Table 3:** Effects of oral treatment with HDP-CDV, ODE-CDV, OLP-CDV or OLE-CDV on mortality of BALB/c mice inoculated intranasally with vaccinia virus-WR.

**Treatment and time (h) of administration^a^**	**Mortality**		***P* value for mortality**	**MDD^b^**	***P* value for MDD**
	**No. of mice that died/total no. infected**	**%**			
Placebo (saline at 24 h)	15/15	100		6.8 ± 0.4	
CDV					
24	0/15	0	<0.001		
48	4/15	27	<0.001	7.8 ± 0.5	0.01
72	0/15	0	<0.001		
Placebo (water at 24 h)	15/15	100		6.8 ± 0.7	
CMX001					
24	2/15	13	<0.001	11.0 ± 4.2	<0.05
48	10/15	67	<0.05	8.0 ± 1.2	<0.01
72	14/15	93	NS	7.4 ± 0.9	0.07
ODE-CDV					
24	0/15	0	<0.001		
48	6/15	40	0.001	8.0 ± 3.0	0.06
72	15/15	100	NS^c^	7.3 ± 0.8	<0.01
OLP-CDV					
24	11/15	73	NS	9.6 ± 1.3	<0.001
48	12/15	80	NS	7.3 ± 1.7	<0.01
OLE-CDV					
24	4/15	27	<0.001	7.5 ± 3.3	NS
48	9/15	60	<0.05	7.4 ± 0.7	<0.01
72	14/14	100	NS	6.5 ± 0.5	NS

Adapted from [18].

a. The animals were treated with 5 mg/kg of compound once daily for 5 days beginning 24, 48 or 72 h after viral inoculation.

b. MDD, mean ± standard deviation day of death.

c. NS, not significant compared to the placebo treated controls.

**Table 4:** Effects of oral treatment with CMX001 on vaccinia virus IHD respiratory infection in mice.

**Compound (mg/kg per day)**	**Treatment days^a^**	**Mortality**		**P-value for mortality**	**Mean day of death^b^**	**P-value for MDD**
		**#dead/#infected**	**Percent**			
Placebo	1–5	10/10	100		6.5 ± 0.5	
CDV^c^ (100)	1	1/10	10	<0.001	17.0 ± 0	
CMX001 (100)	1	0/10	0	<0.001		
(50)	1	0/10	0	<0.001		
(25)	1	2/10	20	<0.001	16.5 ± 0.7	<0.001
(10)	1–5	7/10	70	NS*	10.9 ± 0.7	<0.001
(5)	1–5	7/10	70	NS	10.4 ± 2.6	<0.01
(2.5)	1–5	10/10	100	NS	7.9 ± 0.7	<0.01

Adapted from [19].

a. Starting 24 h after virus exposure.

b. Of mice that died prior to day 21.

c. CDV was given by ip administration.

* NS, not significant when compared to placebo treated controls.

**Table 5:** Effects of oral treatment with CMX001, ODE-CDV, OLP-CDV or OLE-CDV on mortality of BALB/c mice inoculated intranasally with cowpox virus-BR.

**Treatment and time (h) of administration^a^**	**Mortality**		***P* value for mortality**	**MDD^b^**	***P* value for MDD**
	**No. of mice that died/total no. infected**	**%**			
Placebo (saline at 24 h)	15/15	100		9.7 ± 0.6	
CDV					
48	0/15	0	<0.001		
72	5/15	33	<0.001	13.2 ± 3.0	<0.01
Placebo (water at 24 h)	15/15	100		9.3 ± 0.6	
CMX001					
24	6/15	40	0.001	9.5 ± 4.8	NS^c^
48	12/14	86	NS	10.5 ± 3.7	NS
72	7/15	47	<0.01	12.7 ± 3.3	<0.001
ODE-CDV					
24	3/13	23	<0.001	9.3 ± 6.1	NS
48	6/14	43	<0.01	12.7 ± 4.9	0.01
72	7/13	54	0.02	11.6 ± 4.1	0.07
OLP-CDV					
24	12/14	86	NS	11.4 ± 2.5	<0.01
48	4/14	29	<0.001	12.5 ± 3.7	0.09
72	12/14	86	NS	10.3 ± 2.1	0.02
OLE-CDV					
24	8/15	53	<0.01	13.0 ± 6.2	NS
48	5/15	33	<0.001	12.0 ± 3.4	<0.001
72	11/14	79	NS	11.5 ± 4.5	0.02

Adapted from [18].

a. The animals were treated with 6.7 mg/kg of compound once daily for 5 days beginning 24, 48 or 72 h after viral inoculation.

b. MDD, mean ± standard deviation day of death.

c. NS, not significant compared to the placebo treated controls.

**Table 6:** Cytotoxicity and antiviral activity of ST-246 or CMX001 against vaccinia or cowpox virus in human foreskin fibroblast cells.

	**Vaccinia Virus Copenhagen**			**Vaccinia Virus WR**		**Cowpox Virus Brighton**	

Compound	CC_50_ (μM)^a^	EC_50_ (μM)^a^	SI^b^	EC_50_ (μM)^a^	SI^b^	EC_50_ (μM)^a^	SI^b^
ST-246	>100 ± 0	0.05 ± 0.02	>2000	0.1 ± 0.05	>1000	0.48 ± 0.01	>208
CMX001	42 ± 25	0.14 ± 0.09	300	0.13 ± 0.01	323	0.24 ± 0.1	175
CDV	>317 ± 0	29.2 ± 14	>10.9	33 ± 13	>9.6	41.1 ± 4.2	>7.7

Adapted from [24].

a. Values are the mean of 2 or more assays ± standard deviation.

b. Selectivity Index (SI) = CC_50_/EC_50_; CC_50_ (concentration causing cytotoxic effect on 50% of uninfected confluent cells); EC_50_ (effective concentration that reduced plaque formation by 50%).

**Table 7:** Effect of duration of treatment with ST-246 on mortality of BALB/c mice inoculated intranasally with cowpox or vaccinia virus.

	**Cowpox Virus, strain BR**					**Vaccinia Virus, strain WR**				

**Treatment^a^**	**Mortality**		**P-Value**	**MDD^b^**	**P-Value**	**Mortality**		**P-Value**	**MDD^b^**	**P-Value**
	**Number**	**Percent**				**Number**	**Percent**			
5 day duration + 4 h										
Vehicle	15/15	100	---	9.1	---	15/15	100	---	6.1	---
ST-246 100 mg/kg	13/15	87	NS	11.6	0.001	2/15	13	<0.001	3.0	<0.05
5 day duration + 24h										
Vehicle	15/15	100	---	8.6	---	15/15	100	---	6.3	---
ST-246 100 mg/kg	11/15	73	NS	12.4	<0.001	1/15	7	<0.001	3.0	0.08
CDV 15 mg/kg	0/15	0	<0.001	---	---	1/15	7	<0.001	15.0	0.08
7 day duration + 4 h										
Vehicle	15/15	100	---	8.2	---	15/15	100	---	5.7	---
ST-246 100 mg/kg	1/15	7	<0.001	5.0	0.08	3/15	20	<0.001	6.3	NS
7 day duration + 24 h										
Vehicle	15/15	100	---	8.5	---	15/15	100	---	6.3	---
ST-246 100 mg/kg	6/15	40	0.001	9.3	NS	1/15	7	<0.001	11.0	0.09
10 day duration + 4 h										
Vehicle	15/15	100	---	8.3	---	15/15	100	---	6.1	---
ST-246 100 mg/kg	4/15	27	<0.001	8.0	NS	5/15	33	<0.001	10.6	0.06
10 day duration + 24 h										
Vehicle	15/15	100	---	7.9	---	15/15	100	---	6.1	---
ST-246 100 mg/kg	6/15	40	0.001	13.2	<0.01	0/15	0	<0.001	---	---
14 day duration + 4 h										
Vehicle	14/15	93	---	9.1	---	15/15	100	---	5.6	---
ST-246 100 mg/kg	1/15	7	<0.001	3.0	0.09	3/15	20	<0.001	5.3	0.05
14 day duration + 24 h										
Vehicle	15/15	100	---	8.5	---	15/15	100	---	6.7	---
ST-246 100 mg/kg	0/15	0	<0.001	---	---	1/15	7	<0.001	3.0	0.09

Adapted from [23].

a. Mice were treated with durations ranging from 5 to 10 days with treatment beginning from 4 to 24 h post viral inoculation.

b. MDD = Mean Day of Death.

c. NS = Not significant when compared to the appropriate vehicle control.

**Table 8:** Effect of dose and delayed treatment with ST-246 on mortality of BALB/c mice inoculated intranasally with cowpox virus.

**Treatment^a^**		**Mortality**		**P-Value**	**MDD^b^**	**P-Value**
		**Number**	**Percent**			
4 h post inoculation						
Vehicle		15/15	100	---	9.0	---
CDV 15 mg/kg		0/15	0	<0.001	---	---
ST-246	100 mg/kg	1/9	11	<0.001	10.0	NS
	30 mg/kg	5/10	50	0.01	10.2	NS
	10 mg/kg	11/12	92	NS	12.2	<0.01
24 h post inoculation						
Vehicle		15/15	100	---	8.3	---
CDV 15 mg/kg		0/15	0	<0.001	---	---
ST-246	100 mg/kg	4/15	27	<0.001	8.0	NS
	30 mg/kg	6/15	40	0.001	10.5	NS
	10 mg/kg	11/15	73	NS	14.3	<0.001
48 h post inoculation						
Vehicle		15/15	100	---	8.6	---
CDV 15 mg/kg		0/15	0	<0.001	---	---
ST-246	100 mg/kg	1/15	7	<0.001	17.0	0.08
	30 mg/kg	3/15	20	<0.001	14.3	NS
	10 mg/kg	2/15	13	<0.001	11.0	NS
72 h post inoculation						
Vehicle		15/15	100	---	8.6	---
CDV 15 mg/kg		0/15	0	<0.001	---	---
ST-246	100 mg/kg	6/15	40	0.001	16.8	<0.05
	30 mg/kg	6/15	40	0.001	12.2	<0.05
	10 mg/kg	7/15	47	<0.01	13.9	0.001

Adapted from [23].

a. Animals were treated once daily for 14 days beginning 4, 24, 48 or 72 h post viral inoculation.

b. MDD = Mean Day of Death.

c. NS = Not significant when compared to the appropriate vehicle control.

**Table 9:** Effect of combination treatment with ST-246 and CMX001 on mortality of BALB/c mice inoculated intranasally with cowpox virus.

**Treatment^a^**	**Mortality**		**P-value**	**MDD^b^**	**P-value**
	**Number**	**Percent**			
**Vehicle Day 6**	15/15	100	---	10.9 ± 0.6	---
**CDV Day 6**					
25 mg/kg	12/15	80	NS	11.5 ± 3.5	NS
15 mg/kg	9/15	60	0.01	12.8 ± 4.1	NS
5 mg/kg	14/15	93	NS	11.2 ± 3.2	NS
**ST-246 Day 6**					
10 mg/kg	15/15	100	NS	13.5 ± 2.0	0.001
3 mg/kg	12/15	80	NS	13.5 ± 2.4	0.001
1 mg/kg	15/15	100	NS	9.5 ± 0.5	<0.001
**CMX001 Day 6**					
3 mg/kg	15/15	100	NS	9.9 ± 0.9	0.001
1 mg/kg	15/15	100	NS	9.9 ± 1.2	0.001
0.3 mg/kg	15/15	100	NS	10.0 ± 0.8	<0.01
**ST-246 + CMX001 Day 6**					
ST-246 10 mg/kg + CMX001 3 mg/kg	1/15	7	<0.001	11.0 ± 0	NS
ST-246 10 mg/kg + CMX001 1 mg/kg	12/15	80	NS	13.3 ± 3.7	NS
ST-246 10 mg/kg + CMX001 0.3 mg/kg	15/15	100	NS	11.3 ± 1.6	NS
ST-246 3 mg/kg + CMX001 3 mg/kg	12/15	80	NS	12.4 ± 3.9	NS
ST-246 3 mg/kg + CMX001 1 mg/kg	9/15	60	0.01	11.7 ± 2.1	NS
ST-246 3 mg/kg + CMX001 0.3 mg/kg	15/15	100	NS	12.4 ± 1.8	<0.01
ST-246 1 mg/kg + CMX001 3 mg/kg	6/15	40	<0.001	11.8 ± 1.5	NS
ST-246 1 mg/kg + CMX001 1 mg/kg	15/15	100	NS	9.9 ± 1.0	<0.01
ST-246 1 mg/kg + CMX001 0.3 mg/kg	14/15	93	NS	10.5 ± 1.3	NS

Adapted from [24].

a. Animals were treated daily for five days beginning 6 days after viral inoculation.

b. MDD = Mean Day of Death.

c. NS = Not significant when compared to the vehicle control.
